# Supplementary material for: (S)-10-Hydroxycamptothecin Inhibits Esophageal Squamous Cell Carcinoma Growth In Vitro and In Vivo Via Decreasing Topoisomerase I Enzyme Activity
Source: Cancers (Basel). 2019 Dec 6;11(12):1964. doi: 10.3390/cancers11121964 (PMC6966462; doi:10.3390/cancers11121964)
Supplement: Supplementary file 1 [file cancers-11-01964-s001.pdf]

Supplementary Materials: (S)-10-Hydroxycamptothecin Inhibits Esophageal Squamous Cell Carcinoma Growth In Vitro and In Vivo Via Decreasing Topoisomerase I Enzyme Activity

Mengqiu Song, Shuying Yin, Ran Zhao, Kangdong Liu, Joydeb Kumar Kundu, Jung-Hyun Shim, Mee-Hyun Lee and Zigang Dong

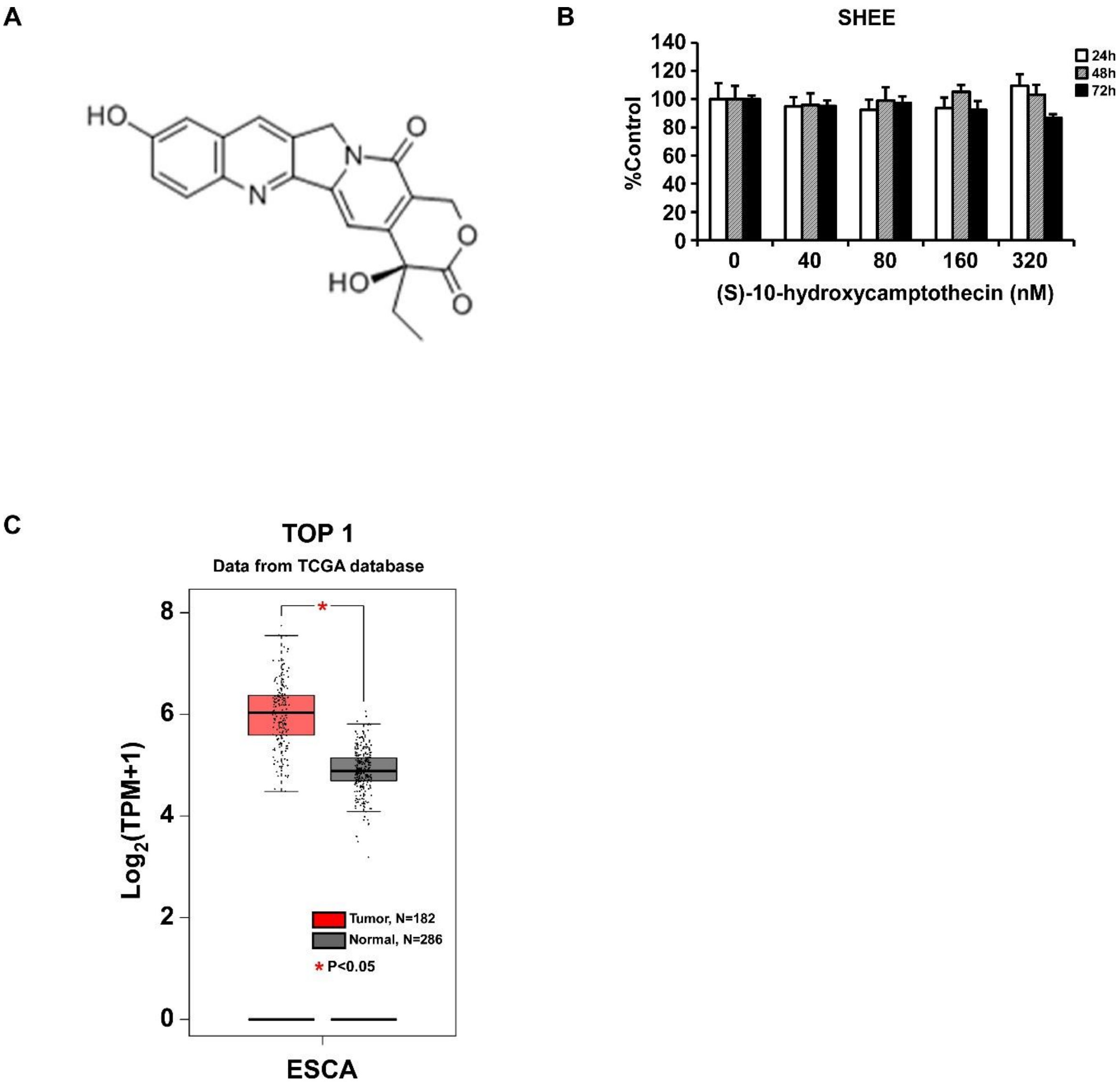

Figure S1. (**A**). The chemical structure of HCPT; (**B**). Cytotoxicity of HCPT on normal esophageal epithelial cell SHEE. (**C**). TOP I gene showed significantly enrichment and amplified in mRNA level in esophageal patient tissues compared to normal tissues in the TCGA data base.

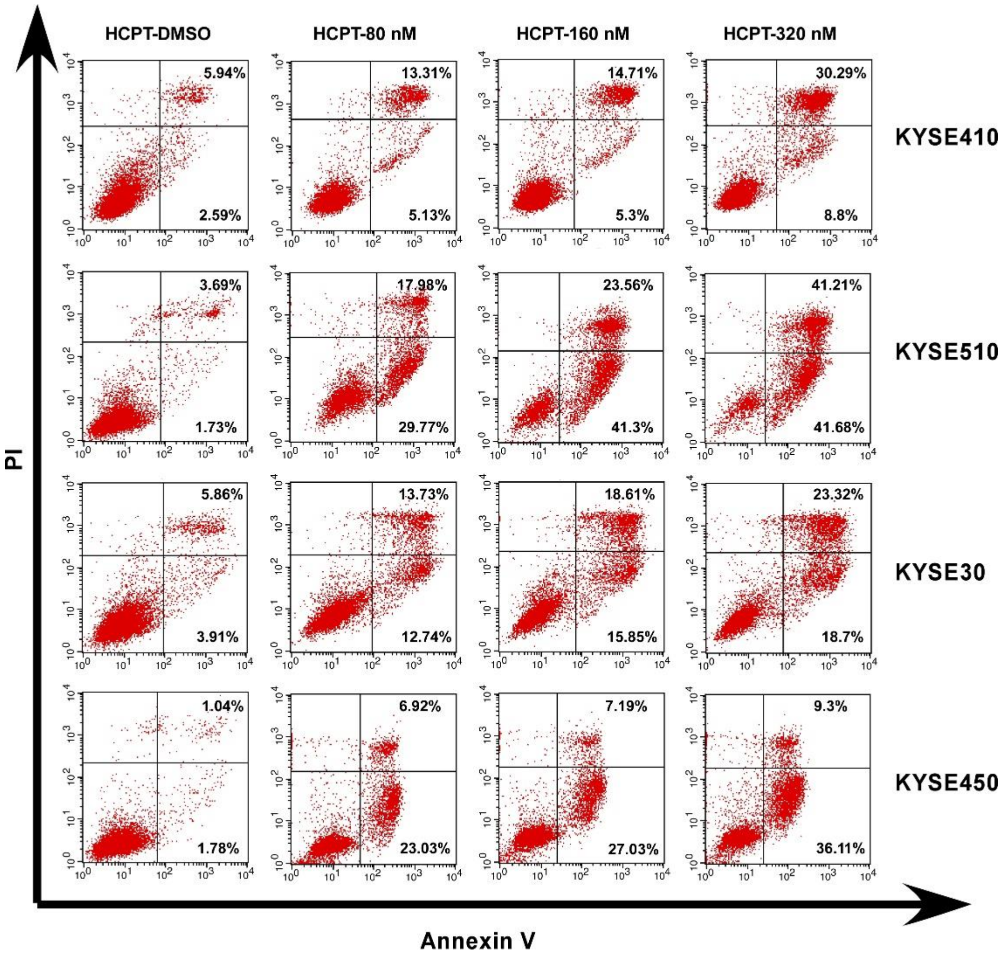

Figure S2. Annexin V and propidium iodide double staining method was used to detect apoptotic cells by flow cytometry.

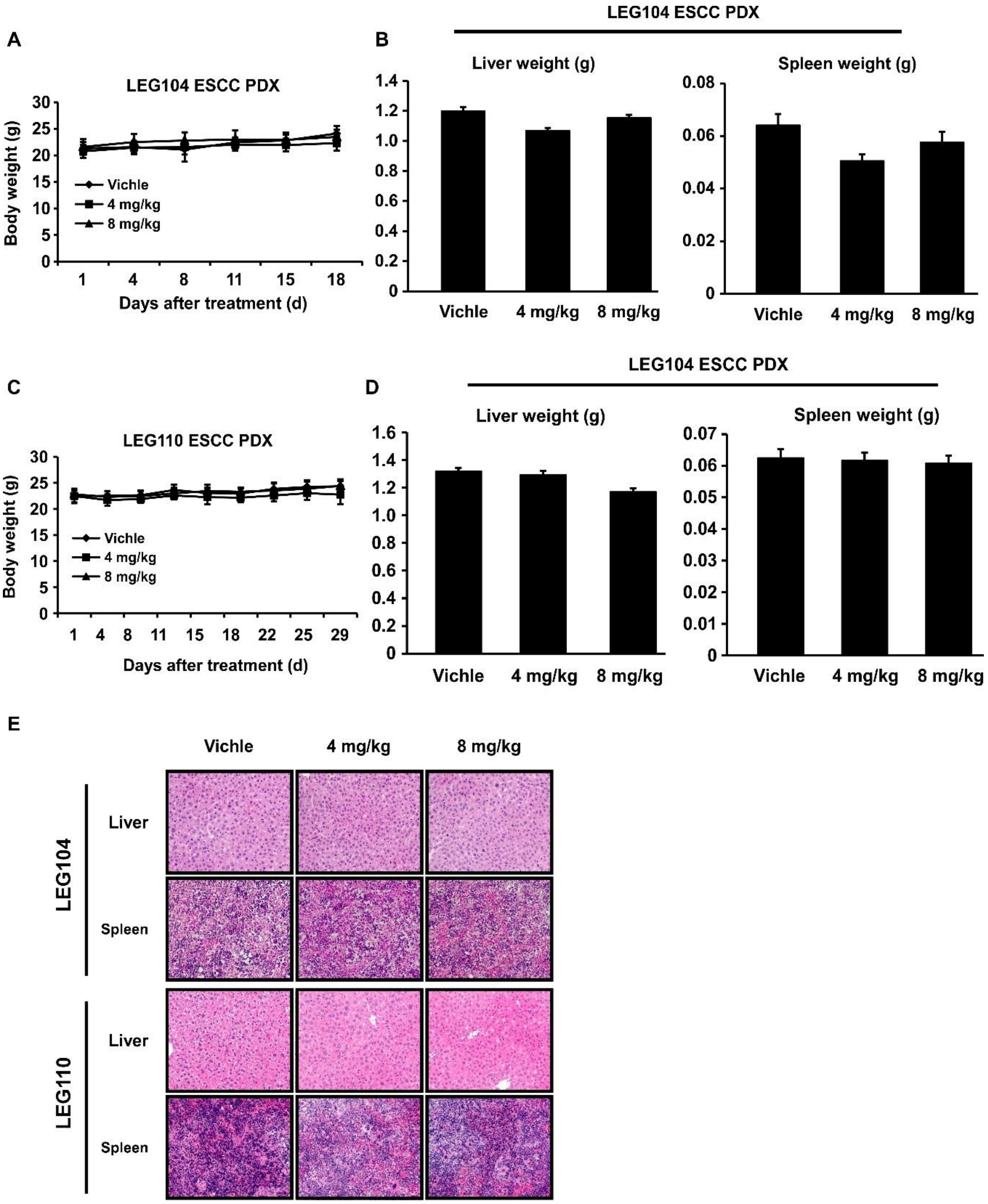

Figure S3. HCPT administration showed no toxicity in PDX animal experiment.

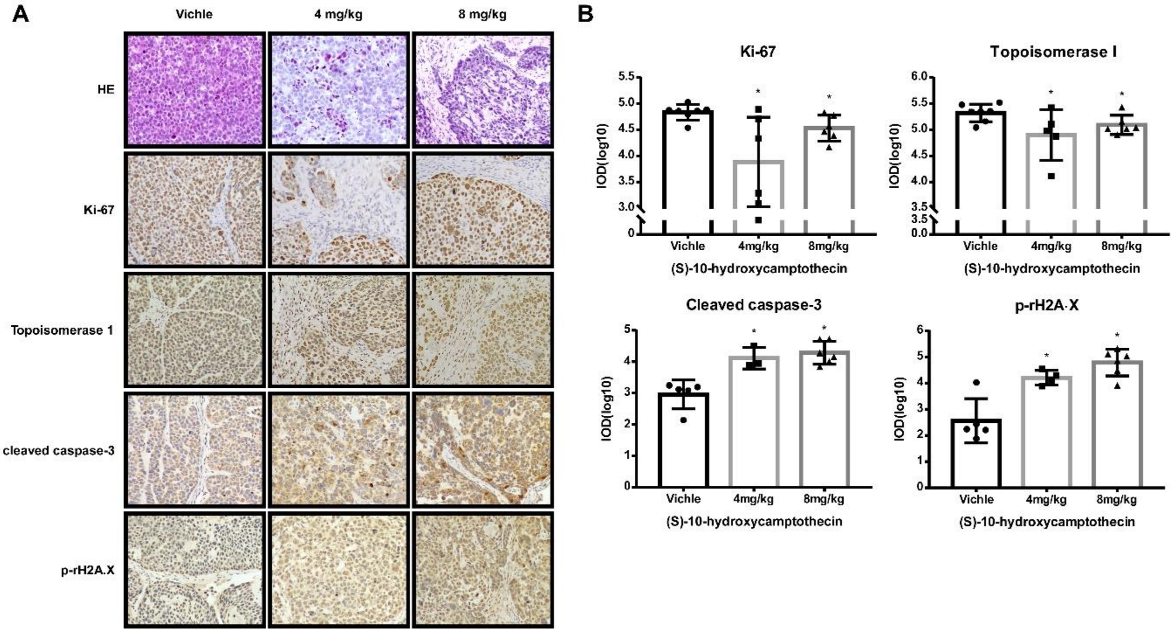

Figure S4. IHC staining in LEG110 PDX tissue specimens (100× magnification).

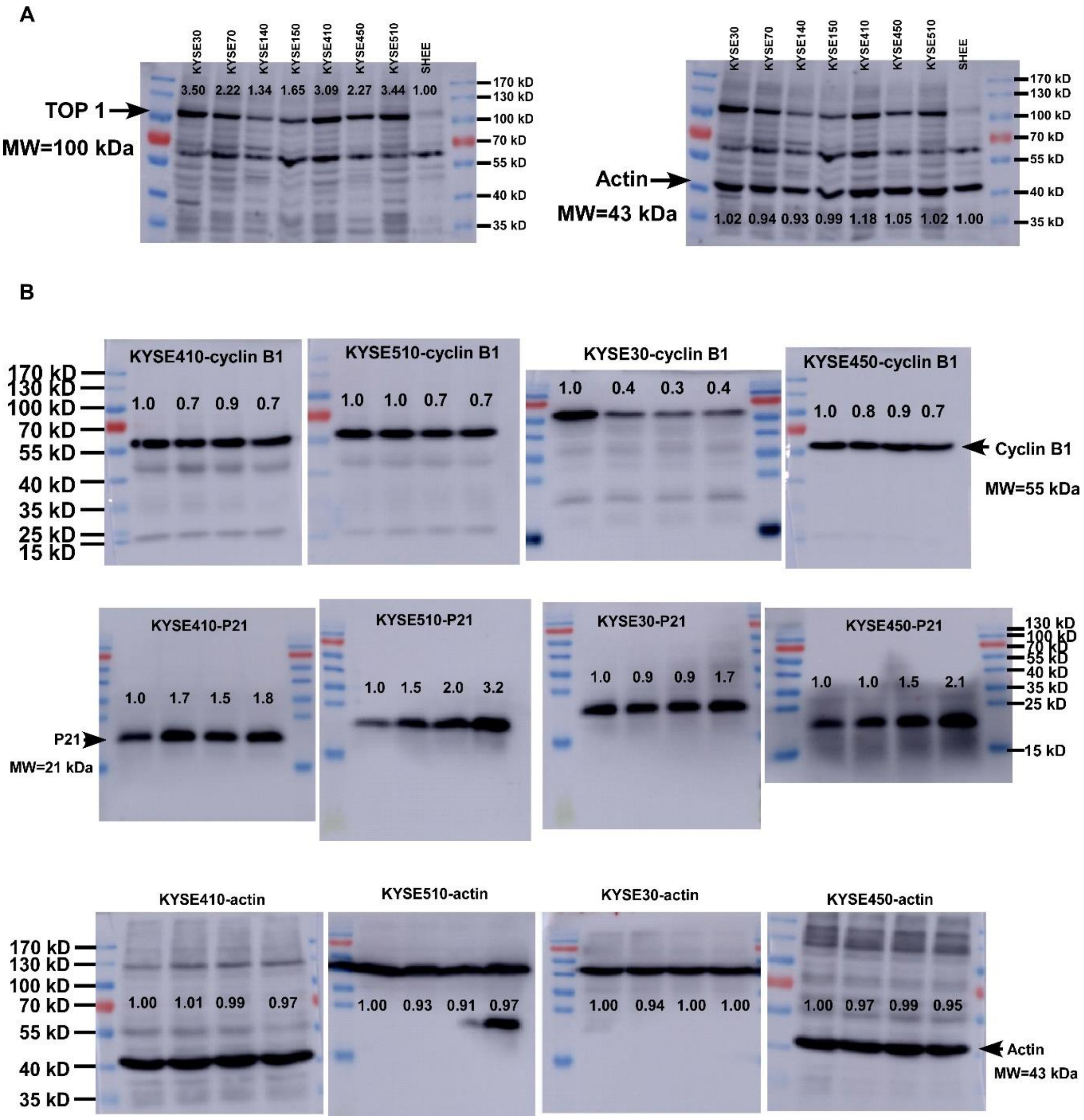

Figure S5. The original western blot data of the expression of TOP I in different cell lines and cell cycle markers.

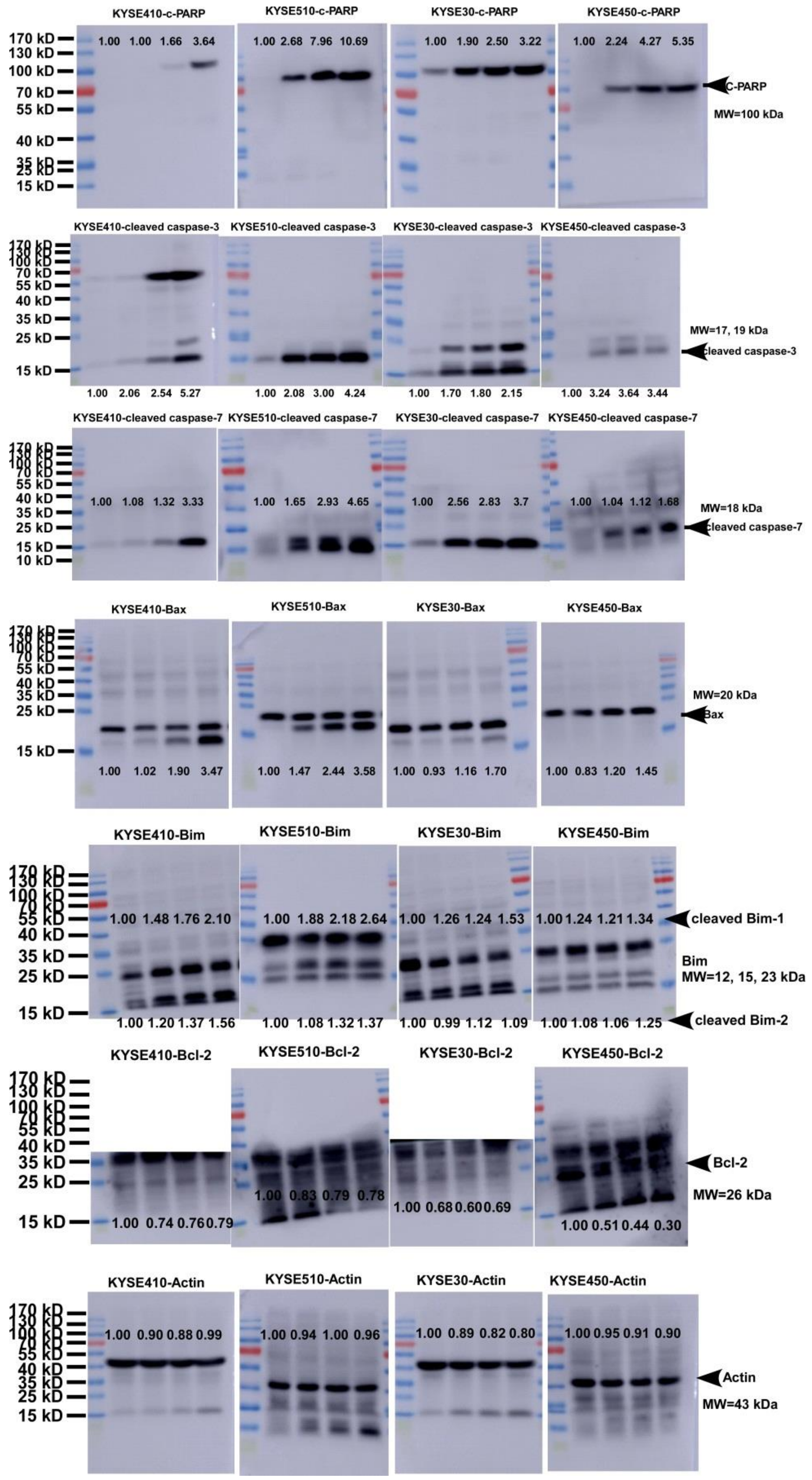

Figure S6. The original western blot data of the expression of apoptosis markers in the four candidate cell lines.

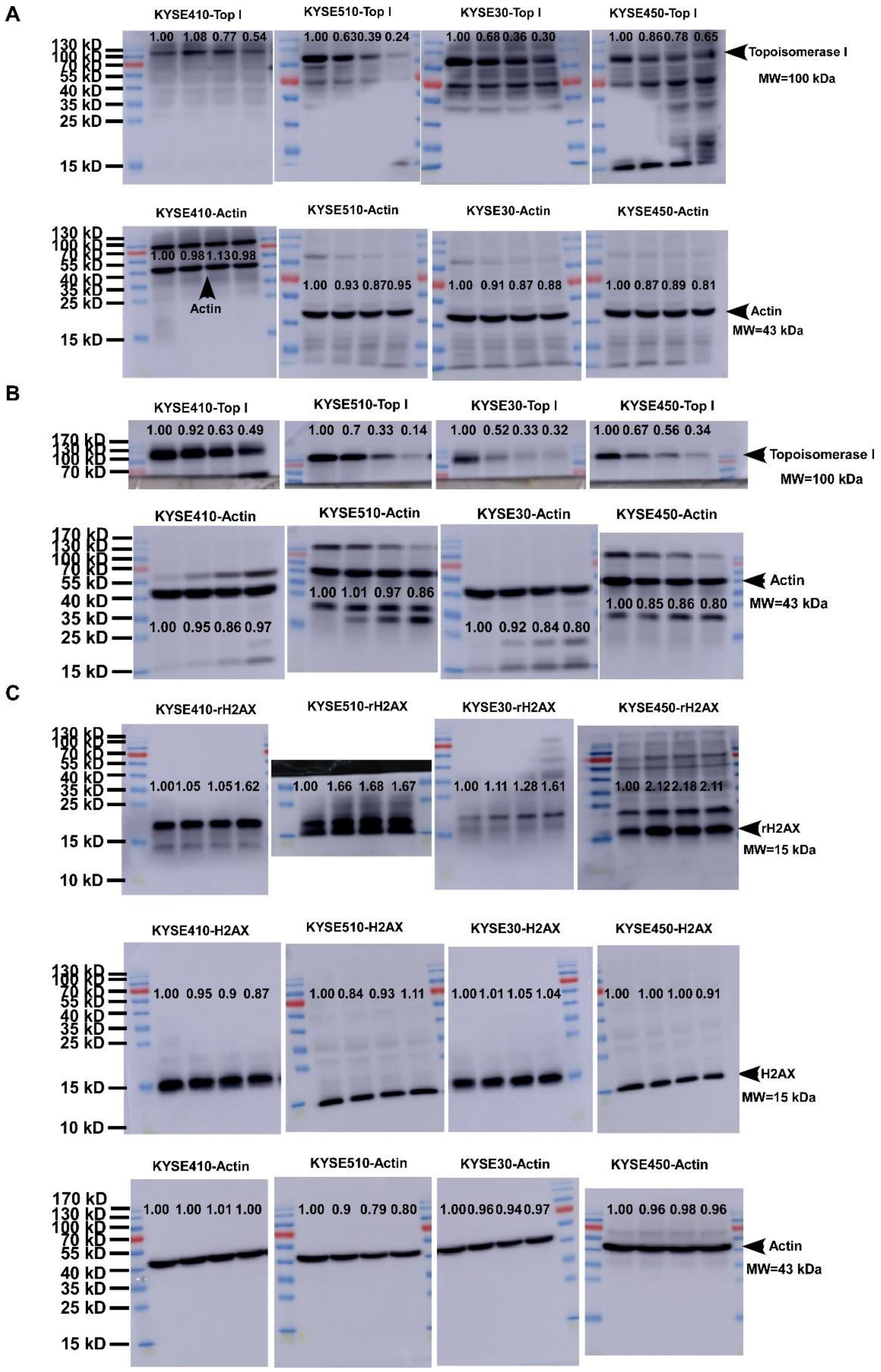

Figure S7. The original western blot data of the expression of TOP I and rH2AX in the four candidate cell lines 48 or 72 h after HCPT treatment.

© 2019 by the authors. Licensee MDPI, Basel, Switzerland. This article is an open access article distributed under the terms and conditions of the Creative Commons Attribution (CC BY) license (http://creativecommons.org/licenses/by/4.0/).
